# Spontaneous Exposure of Mandibular Torus: A Case Report and Surgical Management

**DOI:** 10.1155/crid/8869192

**Published:** 2025-04-29

**Authors:** Priscilla Sergi, Subhi Tayeb, Carlo Barausse, Gerardo Pellegrino, Lorenzo Roccoli, Stefano Ratti, Pietro Felice

**Affiliations:** ^1^Unit of Oral Surgery, Department of Biomedical and Neuromotor Sciences, University of Bologna, Bologna, Italy; ^2^Centre for Clinical and Surgical Experimental and Molecular Anatomy, Department of Biomedical and Neuromotor Sciences, University of Bologna, Bologna, Italy

**Keywords:** bone exposure, case report, histological analysis, mandibular torus, oral exostosis, surgical excision

## Abstract

**Background:** Oral exostoses, including mandibular tori, are benign bony outgrowths that can lead to functional impairments when large. Spontaneous exposure of a mandibular torus is a rare event that requires surgical intervention.

**Case Presentation:** A 33-year-old male presented with bilateral mandibular tori. The right mandibular torus had spontaneously exposed, causing pain during mastication, swallowing, and phonation. Surgical removal of the exposed torus and extraction of a carious tooth were performed under local anaesthesia.

**Conclusion:** The patient recovered without complications, and no recurrence of symptoms was noted during the 1-year follow-up. This case highlights the need for prompt surgical intervention in cases of spontaneous torus exposure and the importance of histological analysis.

## 1. Introduction

Oral exostoses, including mandibular torus and torus palatinus, are benign bony outgrowths originating from the cortical surfaces of the jawbones [1]. Although their occurrence is relatively common in certain populations, spontaneous exposure of these lesions is exceedingly rare and poorly documented in the literature, with minimal consensus on its pathogenesis or clinical management. The rarity of this condition highlights the need for case reports to improve clinical understanding and management [2]. Mandibular torus, typically located on the lingual aspect of the mandible near the premolars and canines, is most often asymptomatic. Histologically, these exostoses consist of mature cortical and trabecular bone, surrounded by periosteum with limited vascularity. While their slow, progressive growth generally halts without intervention, their potential for causing significant functional impairments should not be overlooked [3]. In rare cases, spontaneous bone exposure, as in the present case, can result in debilitating pain and compromised oral function, necessitating surgical intervention.

The etiology of tori remains debated. Traditional hypotheses have focused on mechanical stress from mastication [4], inflammatory agents [5] genetic predisposition, and environmental factors [6]. However, these explanations do not fully account for instances of spontaneous exposure, which is far less understood. Although trauma and inflammation may play a role, no definitive mechanisms have been established. Existing literature primarily addresses general presentations of tori, with limited attention paid to the rare complication of spontaneous exposure. Furthermore, recent advances in imaging and histological analysis have yet to be fully integrated into the understanding of this phenomenon.

Clinically, these exostoses are characterized by a thin, poorly vascularized mucosa covering a nodular, flat, or pedunculated bony prominence, featuring a dense cortical layer and a smaller quantity of spongiosa. Growth is typically slow and progressive, often halting spontaneously [1].

Treatment is only necessary when the exostoses become large enough to cause functional issues, such as impairing speech, swallowing, obstructing airways (leading to sleep apnea), or causing difficulties during general anaesthesia (in cases where nasotracheal intubation is not possible). Other indications include frequent ulcerations over the bony prominence, the need for bisphosphonate therapy, or prosthetic planning for removable partial or complete dentures [7].

When surgical intervention is indicated, the hyperplastic bone is removed under local anaesthesia. General anaesthesia is generally not recommended due to the unnecessary risk it poses to the patient for this type of procedure [8]. The surgery typically involves a marginal intrasulcular incision, as most exostoses (except for the torus palatinus) are located near the gingival margin. The aim is to create adequate visibility while preserving the vascular supply. Flap elevation is performed full-thickness, with careful dissection to avoid tearing the thin mucosa covering the lesion. Once exposed, the lesion is excised using rotary instruments such as fissure burs or piezoelectric devices, with large burs used for bone contouring and smoothing [8].

Postoperative complications may include common signs and symptoms like oedema, hematoma, and mild pain. Postoperative pharmacological treatment includes antibiotics, analgesics, anti-inflammatory medications, and chlorhexidine rinses to promote proper healing [8].

Exposed tori or exostoses may be associated with traumatic or accidental injuries from brushing, hard foods, or inflammatory conditions such as necrotizing stomatitis or osteomyelitis, though spontaneous exposure and sequestration of alveolar cortical bone unrelated to trauma or infection are rare [9, 10].

This case report presents a unique instance of spontaneous mandibular torus exposure in a young adult male, which led to significant functional impairments in mastication, swallowing, and phonation. The case highlights the need for prompt surgical intervention and histological analysis to confirm diagnosis and rule out other pathologies.

## 2. Case Presentation

This case report was prepared in accordance with the CARE (CAse REport) guidelines to ensure completeness and transparency in clinical reporting.

A 33-year-old African American male (ASA II due to smoking 3–5 cigarettes per day) presented with bilateral mandibular tori. He was not taking any medications and reported no known systemic diseases or allergies.

Routine preoperative blood investigations, including complete blood count (CBC), coagulation profile, fasting blood glucose, and liver and renal function tests, were all within normal limits.

He reported severe pain localized to the right mandibular torus (Quadrant IV) during mastication, swallowing, and phonation for 2 weeks prior to consultation.

**Figure 1:**
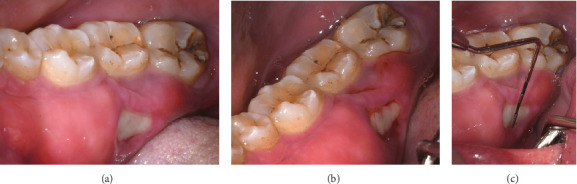
(a–c) Preoperative clinical photographs of the mandibular torus. (a) Intraoral view showing the exposed torus on the lingual aspect of the mandible. (b) Close-up view of the torus, highlighting the inflamed and erythematous mucosa surrounding the exposed bone. (c) Lateral view demonstrating the extent of the torus along the mandibular arch prior to surgical intervention.

**Figure 2:**
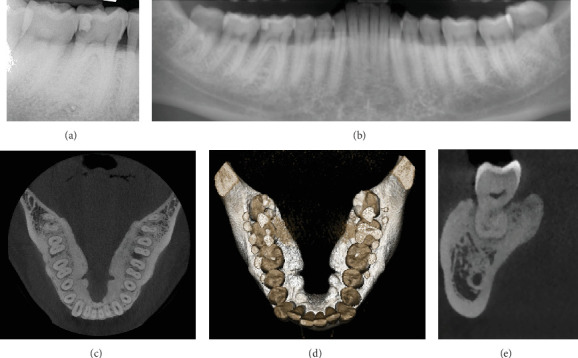
(a–e) Radiographic evaluation of the mandibular torus through various imaging modalities. (a) Periapical radiograph. (b) Panoramic radiograph (orthopantomogram). (c–e) CBCT scans revealing the detailed three-dimensional structure and extent of the torus.

Clinical examination revealed bilateral tori extending from the second molar to the lateral incisor. The right mandibular torus showed 11 mm of mesiodistal bone exposure and 5 mm buccolingual exposure at the lingual aspect of Tooth 47, with surrounding erythematous and inflamed mucosa (Figure 1). Additionally, caries were noted on Teeth 18, 46, and 48, and extrusion of Tooth 38 due to the absence of Tooth 28. Radiographic evaluation (orthopantomogram, periapical, and cone beam computer tomography) did not reveal any cortical discontinuity at the site of bone exposure (Figure 2).

Given the patient's pain and the extent of the bone exposure, surgical removal of the mandibular torus and extraction of the carious Tooth 48 were planned. The treatment was carried out in full compliance with the ethical principles of the Declaration of Helsinki. The patient was treated with respect and dignity, prioritizing their well-being throughout the procedure. Informed consent was obtained, and all measures were taken to protect the patient's rights, health, and confidentiality.

**Figure 3:**
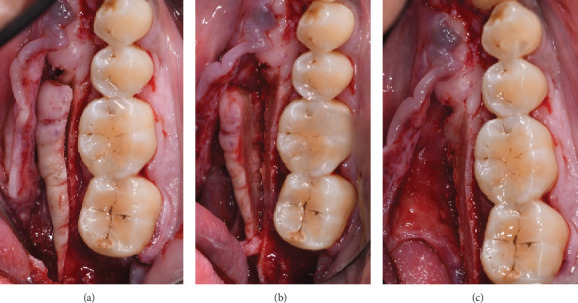
(a–c) Intraoperative photographs. (a) Elevation of the mucoperiosteal flap exposing the mandibular torus. (b) Sectioning of the torus using piezoelectric instruments under sterile saline irrigation. (c) Complete removal of the torus, with the surgical site prepared for final contouring and closure.

The procedure was performed under local anaesthesia. The patient received 875 mg/125 mg amoxicillin/clavulanic acid prophylaxis, followed by an inferior alveolar nerve block using mepivacaine without adrenaline and supplemental infiltration with articaine 1:100,000 adrenaline. A marginal incision was made from Tooth 47 to Tooth 42 with a mesial releasing incision to ensure optimal exposure. The mucoperiosteal flap was elevated with care due to the thin mucosa overlying the torus. Piezoelectric instruments (Mectron, Genova, IT) with sterile saline irrigation were used for the initial osteotomy, followed by chisels to complete the excision. Osteoplasty was performed using a diamond bur to contour the remaining bone (Figure 3).

Wound closure was achieved with 5/0 PGA sutures using vertical mattress stitches at the papillae and simple interrupted sutures at the releasing incision.

Postoperative care included amoxicillin/clavulanic acid (875 mg/125 mg every 8 h for 5 days), ibuprofen (600 mg as needed), and chlorhexidine 0.2% mouth rinses for a week to promote healing.

## 3. Results

Two bone fragments, measuring 3 and 2 cm, corresponding to the molar and canine regions of the mandibular torus, were submitted for histological analysis. The histological analysis revealed mature lamellar bone with areas of focal acute inflammation and fibrosis. These inflammatory changes were interpreted as secondary to chronic mucosal irritation due to the spontaneous exposure of the torus. Immunohistochemistry for MDM2 (Murine Double Minute 2) was negative, supporting the diagnosis of non-neoplastic, inflamed mandibular tori.

**Figure 4:**
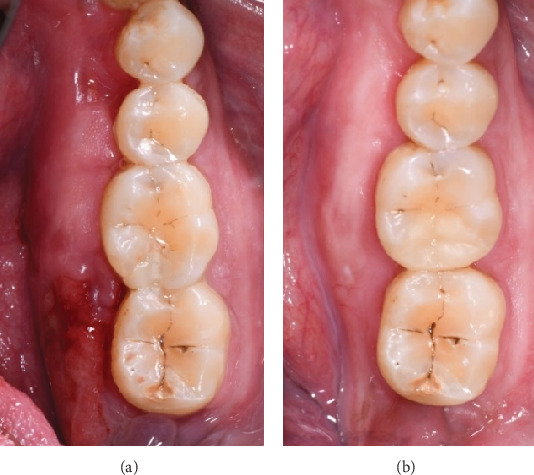
(a, b) Postoperative follow-up. (a) Clinical view at 2 weeks postsurgery showing good healing of the mucosa with no signs of infection or complications. (b) One-year follow-up showing complete healing, with no recurrence of symptoms or bone exposure.

The patient was followed up at 1 week, 2 weeks, 1 month, 6 months, and annually thereafter. Sutures were removed at 2 weeks, and healing was progressing well. At the 1-year follow-up, no further bone exposure was noted, and the patient remained asymptomatic (Figure 4). The contralateral mandibular torus showed no signs of progression or symptoms.

## 4. Discussion

Spontaneous exposure of mandibular tori is an infrequent phenomenon, with limited cases documented in the literature. Most studies and reviews focus on the general characteristics, formation, and clinical presentation of tori, but there is a notable lack of comprehensive data on spontaneous exposure. Of the few cases reported, the pathogenesis remains unclear, with suggested etiological factors including masticatory stress, localized trauma, genetic predisposition, and environmental influences.

In this particular case, the patient had undergone general anaesthesia for an unrelated surgical procedure a month prior to presenting with pain associated with the exposed torus. While the patient did not report any immediate airway management complications, it is conceivable that manipulations during intubation or positioning during surgery may have contributed to trauma of the mandibular torus, leading to its exposure. During orotracheal intubation, pressure from the laryngoscope blade on the floor of the mouth, or hyperextension of the neck, may exert mechanical forces directly onto the lingual cortical plate, particularly in the presence of prominent tori [11]. Although such a hypothesis is plausible, the literature lacks specific data linking general anaesthesia with spontaneous torus exposure. A handful of reports discuss the difficulty of intubation in patients with large tori, particularly in the palatal region, where modified intubation techniques such as the use of laryngeal mask airways are necessary [12]. These reports underscore the challenges posed by large tori during airway management but do not provide definitive evidence of trauma-induced exposure [13].

Given this lack of data, it is essential to consider alternative factors in this case. Interestingly, Sonnier and Horning described three cases of spontaneous cortical bone exposure and sequestration associated with mandibular tori, suggesting that chronic mechanical irritation, thin overlying mucosa, and localized vascular compromise may predispose to bone exposure in anatomically vulnerable regions [14].

Masticatory stress, which has been previously implicated in the development of tori, could have played a role in the exposure, especially considering the location and size of the torus. Repeated occlusal loading may result in microtrauma or ischemic changes in the thin, poorly vascularized mucosa covering the torus, ultimately compromising its integrity over time. Additionally, localized inflammatory processes, such as gingival or mucosal inflammation, may have weakened the overlying tissue, increasing the susceptibility to exposure. However, these mechanisms remain speculative and require further investigation. To date, very few reports have addressed similar cases of spontaneous exposure, and those available often lack histological confirmation or detailed surgical follow-up, making direct comparison difficult. Nevertheless, the combination of anatomical, mechanical, and inflammatory factors remains the most plausible multifactorial explanation.

Histopathological analysis revealed mature lamellar bone with focal areas of acute inflammation and fibrosis. Although these findings differ from the classical description of mandibular tori—typically consisting of compact bone with osteocytes and minimal connective tissue reaction—this discrepancy is likely due to the spontaneous exposure and chronic irritation of the lesion. Similar reactive changes have been observed in rare cases of exposed or ulcerated tori. For example, Gombra et al. [15] described a typical torus mandibularis composed of mature lamellar bone without inflammatory changes, while Valentin et al. [16] reported histological features including interosseous vascular spaces and recurrence under mechanical stress. In our case, the presence of inflammation likely reflects a secondary tissue response rather than a deviation from the intrinsic histology of the torus. Furthermore, although not commonly employed for such lesions, MDM2 immunohistochemistry was included in the diagnostic work-up to exclude low-grade central bone tumors, given the unusual clinical presentation involving spontaneous exposure, pain, and soft tissue inflammation. The absence of MDM2 expression confirmed the non-neoplastic nature of the lesion.

The rarity of spontaneous mandibular torus exposure highlights the need for long-term clinical follow-up. In this case, the patient was asymptomatic after 1 year of follow-up, with no recurrence of symptoms or further exposure of the contralateral torus. This outcome aligns with previous studies that suggest surgical removal is curative when performed properly, and that recurrence rates are low if the lesion is adequately excised and monitored. Furthermore, conservative management should be reserved for asymptomatic tori, as surgical intervention is only necessary when complications arise.

In summary, this case contributes to the limited body of literature on spontaneous mandibular torus exposure and underscores the importance of individualized treatment planning. Future research should focus on identifying specific risk factors and mechanisms behind torus exposure as well as optimizing surgical and nonsurgical management strategies.

## 5. Conclusion

Spontaneous exposure of mandibular tori is a rare clinical occurrence that can lead to considerable functional impairment. In the present case, surgical excision under local anaesthesia resulted in complete symptom resolution, with no recurrence at the 1-year follow-up. Histopathological evaluation was essential to confirm the diagnosis and exclude other pathological entities. Prompt surgical management is advised in similar cases, followed by structured long-term follow-up to monitor healing and detect potential recurrence.

## Data Availability

The data used to support the findings of this study are available from the corresponding author upon request.
